# Case for diagnosis. Infiltrated areas on the trunk^☆^^☆☆^

**DOI:** 10.1016/j.abd.2019.12.007

**Published:** 2020-06-25

**Authors:** Larissa Daniele Machado Góes, Juliana Alves Scrignoli, Patrícia Morais, Carolina Talhari

**Affiliations:** a Tropical Dermatology Clinic, Fundação de Dermatologia Tropical e Venereologia Alfredo da Matta, Manaus, AM, Brazil; b Rheumatology Service, Fundação Hospital Adriano Jorge, Manaus, AM, Brazil; c Dermatopathology Service, Fundação de Dermatologia Tropical e Venereologia Alfredo da Matta, Manaus, AM, Brazil

**Keywords:** Leprosy, Leprosy, multibacillary, Panniculitis

## Abstract

Leprosy is an infectious disease with chronic evolution, caused by *Mycobacterium leprae*, an acid-fast bacillus that mainly affects the skin and peripheral nervous tissue. Many of the clinical manifestations of leprosy can mimic connective tissue diseases. The authors present the case of a 49-year-old woman who had been treated for four years for systemic lupus erythematosus in a rheumatological service. Skin biopsy of a plaque on the inguinal region was compatible with borderline lepromatous leprosy associated with a type 1 lepra reaction. The patient is undergoing treatment with multibacillary multidrug therapy, showing clinical improvement.

## Case report

**Figure 1:**
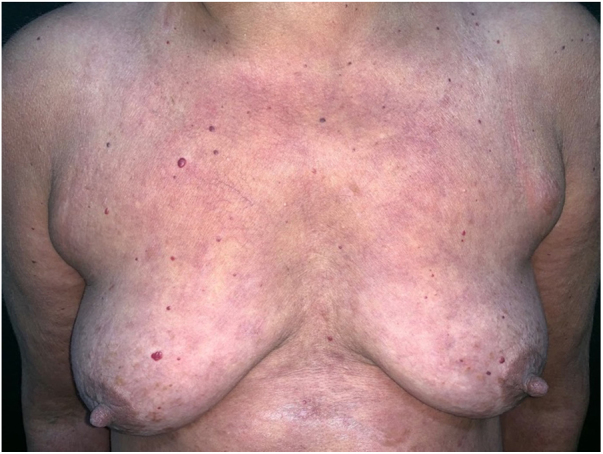
Erythematous plaques on the trunk.

**Figure 2:**
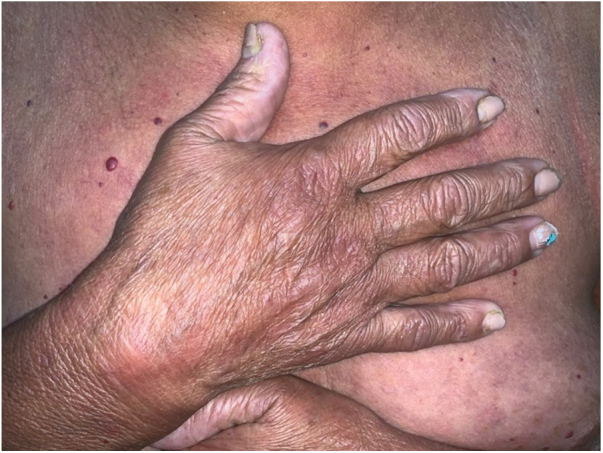
Erythematous plaques on the back of the hand and infiltration of the fingers.

A 49-year-old woman reported pain in the interphalangeal joints and lower limbs, a skin rash, and alopecia; all symptoms persisted for four years. She was being treated for systemic lupus erythematosus with hydroxychloroquine, azathioprine, methotrexate, and prednisone. On physical examination, she presented generalized erythematous plaques, and infiltrated areas on the trunk, on the ears, and fingers, as well as diffuse non-healing alopecia (Figure 1, Figure 2).

She presented 24-hour proteinuria within normal limits, positive ANA (1:320, centromeric dotted nuclear pattern), positive anti-cardiolipin IgM antibodies, and anti-SM and anti-double helix DNA, both negative.

### What is your diagnosis?

a) Subacute lupus
b) Dermatomyositis
c) Lepromatous leprosy
d) Diffuse eosinophilic fasciitis

She also presented paresthesia of the lower limbs, and thickening and pain on palpation of the left ulnar and left external sciatic popliteal nerves.

**Figure 3:**
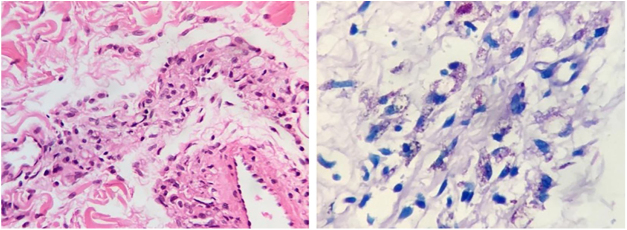
(A), Perivascular and periadnexal inflammatory infiltrate composed of lymphocytes, histiocytes, plasma cells, and epithelioid cells (Hematoxylin & eosin, ×40); (B), Fragmented, granular, isolated acid-fast bacilli, also grouped in globi (Wade, ×40).

**Figure 4:**
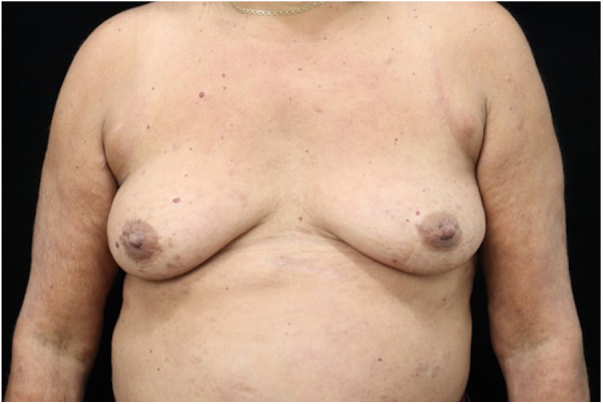
Residual hyperchromic patches on the trunk and upper limbs after 30 days of treatment with multidrug therapy and prednisone.

The bacilloscopy of the ear and two trunk lesions was positive (smear index: 3.33). Histopathology of the plaque in the inguinal region showed an epidermis with hyperkeratosis; in the dermis, edema and a perivascular, periadnexal, and interstitial mild inflammatory infiltrate composed of lymphocytes, histiocytes, plasmocytes, epithelioid cells, and sparse giant Langhans cells, involving the subcutaneous tissue, adipocytes and septa. Wade staining showed a large number of intracellular acid-fast bacilli, grouped in globi, fragmented and granular (Fig. 3A and 3 B). PAS staining did not show thickening of the basal membrane, and no mucin was observed in the Alcian blue staining. In light of the histopathological alterations, a diagnosis of borderline lepromatous leprosy was made in association with type 1 lepra reaction. The patient denied having undergone previous treatment for leprosy and reported that a son-in-law had received anti-leprosy treatment. Multibacillary multidrug therapy and prednisone 60 mg/day were initiated for treating the symptoms and neural lesions. After 30 days of treatment, the patient presented significant improvement (Fig. 4).

## Discussion

Leprosy is a chronic contagious infectious disease, caused by *Mycobacterium leprae*, an acid-fast bacillus, with tropism for Schwann cells and the reticuloendothelial system. The bacillus mainly affects the skin and peripheral nervous tissue.^1^ The ability of *M. leprae* to regulate cytokine production and induce Th1 or Th2 responses contributes to the variety of clinical presentations of the disease.^2^

Rheumatological manifestations are observed in 64% to 77% of patients with multibacillary leprosy, especially when presenting lepra reactions.^3^ Rheumatological symptons were observed in 41.8% of the patients with lepromatous leprosy; in 23.6% of those with tuberculoid borderline; in 18.2% of those with borderline forms; and in 16.4% of those with borderline lepromatous type.^4^

Positive ANA can be observed in 3% to 34% of leprosy patients. Titers are generally low, and the speckled and homogeneous patterns are the most common.^5^ Rheumatoid factor, anti-CCP, ANCA, anti-cardiolipin, and antiphospholipids have been observed in varying percentages in patients with the different forms of leprosy.^6^, ^7^, ^8^ Non-scarring alopecia, especially in patients with the lepromatous leprosy, has already been described.^9^

The different clinical manifestations of leprosy pose a diagnostic challenge, since they can mimic other diseases, including autoimmune disorders. The difficulty of diagnosing leprosy early can increase the chance of transmission and cause deformities secondary to peripheral nerve involvement.^10^

## Financial support

None declared.

## Authors' contributions

Larissa Daniele Machado Góes: Elaboration and writing of the manuscript; critical review of the literature; critical review of the manuscript.

Juliana Alves Scrignoli: Approval of the final version of the manuscript; conception and planning of the study; elaboration and writing of the manuscript; obtaining, analyzing, and interpreting the data; effective participation in research orientation; intellectual participation in propaedeutic and/or therapeutic conduct of studied cases; critical review of the literature; critical review of the manuscript.

Patrícia Morais: Statistical analysis; approval of the final version of the manuscript; conception and planning of the study; elaboration and writing of the manuscript; obtaining, analyzing, and interpreting the data; effective participation in research orientation; intellectual participation in propaedeutic and/or therapeutic conduct of studied cases; critical review of the literature; critical review of the manuscript.

Patrícia Morais: Statistical analysis; approval of the final version of the manuscript; conception and planning of the study; elaboration and writing of the manuscript; obtaining, analyzing, and interpreting the data; effective participation in research orientation; intellectual participation in propaedeutic and/or therapeutic conduct of studied cases; critical review of the literature; critical review of the manuscript.

## Conflicts of interest

None declared.
